# A Novel Atraumatic Tourniquet Technique for Excessive Bleeding during Cesarean Sections

**DOI:** 10.1155/2017/7171520

**Published:** 2017-01-09

**Authors:** Baris Buke, Emre Canverenler, Hatice Akkaya, Fuat Akercan

**Affiliations:** ^1^Department of Obstetrics and Gynecology, Kayseri Education and Research Hospital, Kayseri, Turkey; ^2^Department of Obstetrics and Gynecology, Medical Park Uşak Hospital, Uşak, Turkey; ^3^Department of Obstetrics and Gynecology, Ege University Hospital, İzmir, Turkey

## Abstract

*Objective*. Controlling excessive bleeding in cesarean sections which may cause a life-threatening event even under well-prepared conditions. We used a novel atraumatic tourniquet technique to temporary arrest blood flow through the uterine and ovarian vessels and compare with other techniques. Toothless vascular clamps were used as clamp.* Methods*. Tourniquet technique performed postpartum hemorrhage (PPH) cases (19 out of 37) were compared with 18 other cases with PPH.* Results*. The difference between preoperative and postoperative hemoglobin values was significantly lower in the study group as well as the number of blood products needed during and after surgery.* Conclusions*. This technique not only prevented massive bleeding from the uterus but also allowed physicians time to consider the necessity of further interventions.

## 1. Introduction

Although cesarean sections (CSs) can often be done without problems, profuse bleeding is evident in particular cases. There are several possible reasons that cause excessive bleeding. Bleeding may be from the anterior abdominal wall, due to placental invasion abnormalities or from the entire uterus due to uterine atony. Our technique is useful in both types of uterine bleeding.

Bleeding from the uterus can be from the incision or placental site. Incision site concerns can be thick, lower segment, coagulopathy, extension of incisions that involve uterine vessels, and classical CS problems. Placental site concerns can be atonic uterus, retained placental tissues, and abnormal placental implantation [1].

Once atonic hemorrhage has developed, the management algorithm is the same as after a vaginal delivery. Cesarean section actually provides the advantage of direct compression of the uterus. When compression is effective, it can be ascertained directly by observing blood welling up or the lack thereof. The first step is to gently massage the uterine fundus; if it remains flabby, bimanual compression can be used. Simultaneously, oxytocics should be administered as well as methergine and prostaglandin F2*α*. If these measures fail, one must then consider surgical steps in a sequential manner [2].

Placenta accreta (PA) is another cause of peripartum and postpartum hemorrhage, which may involve maternal morbidity and mortality. The incidence of PA increases with placenta previa (PP), especially with prior CSs [3]. Even with antepartum diagnosis and blood transfusion preparation for CS, it is not uncommon for a patient to be at risk during this kind of delivery due to massive bleeding. Many hemostatic techniques, such as bilateral uterine and internal iliac artery ligation [4], hypogastric and uterine arterial embolization including balloon occlusion [5], interrupted circular sutures such as Hayman and B-Lynch procedures [6, 7], stepwise uterine devascularization [8], and argon beam coagulation techniques [9] have been attempted to minimize blood loss. However, excessive bleeding during CS is still rare and panic only makes it more difficult for obstetrical physicians to select the relevant procedure.

High quality and expensive equipment are only available in tertiary centers. There is a need for a simple hemostatic technique that any physician can do for appropriate decision-making, because rapid onset of profuse bleeding makes it difficult to decide whether further operations such as hysterectomy should be performed.

We have devised a simple method to attain hemostasis after fetus extraction, especially in cases with profuse bleeding. We applied this technique to nineteen cases with unusual bleeding and compared them with eighteen prior cases in which the technique had not yet been implemented.

## 2. Materials and Methods

This retrospective cohort study included cases of excessive bleeding during CS, who gave birth between December 2013 and February 2015 at Ege University Hospital. The study group was composed of postpartum hemorrhage (PPH) cases, in which a novel atraumatic tourniquet technique was used. The control group was composed of well-selected, matched patients who experienced PPH. Women who had coagulopathy disorders, lower uterine segment tears extending to the vagina, and bleeding due to an injured artery were excluded from the study. Demographic and obstetric features were recorded on a review chart with surgical and pregnancy follow-up records in Ege University's patient system. These include maternal age, gestational age at delivery, and cause of PPH and surgical method of choice. Peripartum features include requirement for massive transfusion, number of packed red blood cells (PRBCs), and fresh frozen plasmas (FFP) used during and after surgery. This tourniquet technique was used in March 2014 in emergency surgery for a PP case along with PA.

PP with or without PA and uterine atony were the main cause of PPH in this study, as well as in control groups. It is related to increased maternal mortality and morbidity, so the presence of accreta is of great importance. If suspicion of PA is high, we prefer to open the abdomen through a longitudinal infraumbilical incision to facilitate proper exposure. However, if we encounter a case of atonic uterus, it means that the abdomen has already been opened transversely.

In cases of PA, we preferred to deliver the baby via fundal uterine incision. The uterine incision should be located away from dilated vasculature. Following the delivery of the baby, we exteriorized the uterus to be able to apply our atraumatic tourniquet technique and make the following procedures. The uterovesical fold of the peritoneum was then opened and the bladder was gently pushed down as low as possible.

**Figure 1:**
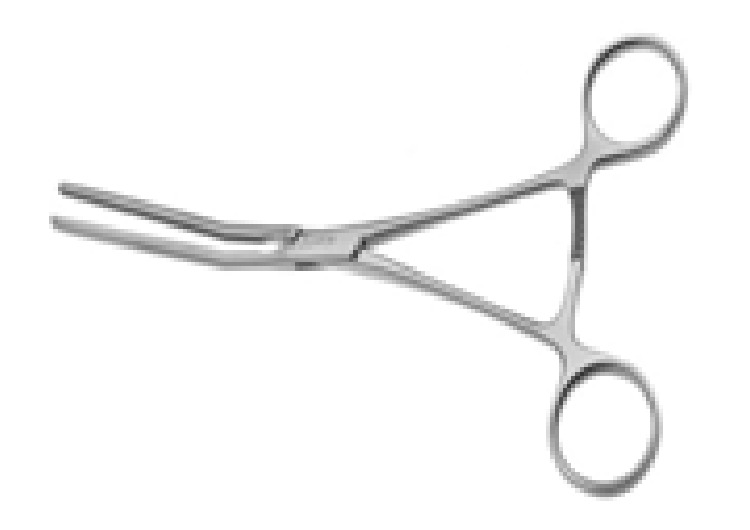
Atraumatic DeBakey vascular clamp.

**Figure 2:**
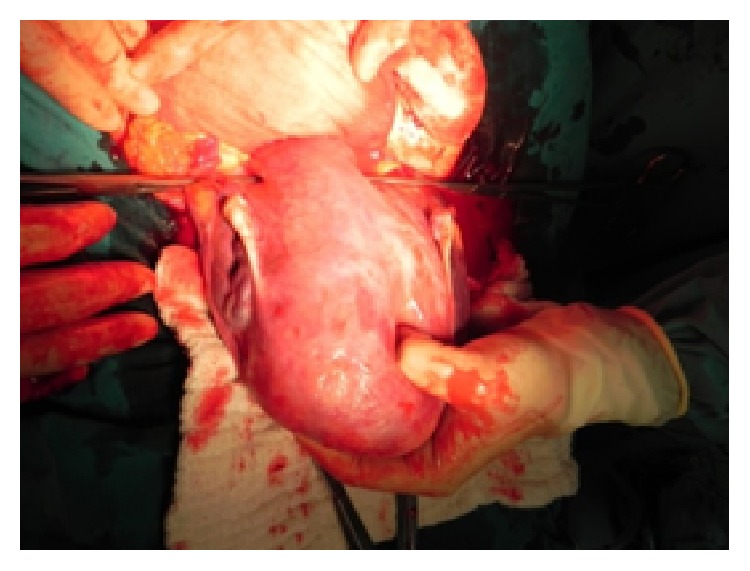
Insertion of clamps.

However, it is not obligatory to push the bladder before the procedure. The surgeon and assistant palpate the ureters with both hands in the two sheats of broad ligament to try to hear a “click” sound, which comes from the ureter sliding out between both fingers. After confirming that the ureters are not in the surgical site, an atraumatic vascular DeBakey clamp (Figure 1) is applied widely on both the infundibulopelvic ligament and the uterine arteries (Figure 2). Thus, two-sided blood supply of the uterus is significantly reduced, which provides time for planning other surgical procedures in a less bloody surgical site. If an avascular state of the surgical site is needed for a prolonged period, while preparing for a blood transfusion or waiting until an experienced surgeon is available, the clamps are opened and closed intermittently for a maximum of 10 minutes to sustain blood supply.

Statistical analysis was performed using Statistical Package for Social Sciences version 21.0 (SPSS; Chicago, IL, USA). The Kolmogorov-Smirnov normality test was applied to all variables. The results were expressed as the mean SD (normally distributed variables) or median with minima and maxima (nonnormally distributed variables). Significant statistical differences involve the atraumatic DeBakey vascular clamp as well.

## 3. Results

The study population included 37 cases of PPH, in which 19 used our technique. We compared data from these 19 patients to 18 other patients with PPH the previous year (Group 2). The most important contributing factors to PPH included PA in 56.8% of all cases (21 out of 37), PP alone in 16.2% of cases (6 out of 37), placenta percreta in 13.5% (5 out of 37), and uterine atony in 13.5% (5 out of 37).

The surgical method of choice was conservative management in 48.6% (18 out of 37) of the entire group, whereas, in other cases, hysterectomy (19 out of 37) was the chosen route. There were no statistically significant differences between groups regarding obstetric and demographic features.

The difference between preoperative and postoperative hemoglobin values was significantly lower in (Group 1), the study group (*p* = 0.02), as well as the number of PRBC and FFP needed during and after surgery (*p* = 0.001, *p* < 0.001, resp.). The peripartum features of the groups are summarized in Table 1.

## 4. Discussion

This research aimed to define a novel atraumatic clamp tourniquet technique, which was proposed in order to lower postpartum hemorrhage in cases of PPH and assess its effectiveness. The results of our study reveal that both the decline in hemoglobin levels and the need for RBC and FFP were reduced in cases for which this new tourniquet technique was used.

To minimize the amount of bleeding in cases of PPH, especially in cases of PP and PA, a few tourniquet techniques were decided in advance. Ikeda et al. were the first to define a tourniquet technique in 2005 [10]. Given this methodology, an assistant grips the uterine cervix and the infundibulopelvic ligaments with both hands to shut off blood circulation from uterine and ovarian vessels. The assistant adjusts manual pressure simultaneously, while gripping the cervix to halt any active bleeding from the uterus. This maneuver can help the surgeon find more time to make a decision, but that only inhibits further intervention like uterine compression sutures, ligation of the vessels, and additional steps in the hysterectomy.

If the tourniquet maneuver is needed for an extended time period, the authors replaced the hand girdle with a rubber tube. It is obvious that, for further interventions, this maneuver will be necessary in most cases. When an immediate hysterectomy must be done, the tourniquet maneuver or tube is replaced by a separate rubber tube tourniquet on the uterine vessels, which includes clamping of the infundibulopelvic ligaments. It should be noted that each of the rubber tube replacements carries a risk of hemorrhage due to possible damage of the vessels, while also making a hole in the broad ligament. Also, such an intervention can take more than 5 minutes, which is an extended period of time in cases of excessive bleeding with PPH.

In another tourniquet technique, described by Tammam et al. in 2012, a Foley's catheter NO. 16 or 18F was placed by surrounding it around the infundibulopelvic ligament, which is long and relaxes in pregnancy, posteriorly at the level of the uterosacral ligament and tied anteriorly at 3 cm to the CS incision with meticulous avoidance of tubal involvement. This method seems feasible and effective at first, but many PPH cases also involve PA, which can extend to the bladder. In these situations, the Foley catheter will slide off and not surround the vessels or bed of the placenta. So this technique will be effective in cases in which the cause of PPH is localized to the uterus and does not extend to the bladder or other organs [11].

The atraumatic clamp tourniquet technique has many advantages over the previously described methods. It is easy to apply, regardless of the size of the hospital. The most critical advantage is the short time involved; it takes only 30 seconds to apply the technique on both sides of the uterus. In addition, the operator does not need to open a hole in the broad ligament. Thus, there is no risk of damage to the vessels located in the broad ligament. It is also viable to loosen and tighten the clamps to maintain uterine and ovarian vitality.

We find this technique to not be therapeutic; this tourniquet allows the physician to plan for further interventions by minimizing blood loss. It is clear that by reducing blood loss, we also reduce maternal morbidity and mortality. In this sense, this is an effective method that looks promising for PPH cases.

## Figures and Tables

**Table 1:** Peripartum features of groups.

	Group 1 (mean ± SD)	Group 2 (mean ± SD)	*p* value
Decline in Hb values (g/dl)	2.1157 ± 1.30565	3.5777 ± 1.29231	**0.002**
Transfusion of red blood cells (units)	1.16 ± 1.167	4.61 ± 3.760	**0.001**
Transfusion of fresh frozen plasma (units)	0.26 ± 0.653	2.33 ± 2.223	**<0.001**
